# Retraction: Yttrium- and zirconium-decorated Mg_12_O_12_–X (X = Y, Zr) nanoclusters as sensors for diazomethane (CH_2_N_2_) gas

**DOI:** 10.1039/d5ra90038g

**Published:** 2025-04-03

**Authors:** Terkumbur E. Gber, Hitler Louis, Obinna C. Ngana, Ismail O. Amodu, Ernest E. Ekereke, Innocent Benjamin, Stephen A. Adalikwu, Adedapo Adeyinka

**Affiliations:** a Computational and Bio-Simulation Research Group, University of Calabar Calabar Nigeria louismuzong@gmail.com; b Department of Pure and Applied Chemistry, Faculty of Physical Sciences, University of Calabar Calabar Nigeria; c Department of Mathematics, Faculty of Physical Sciences, University of Calabar Calabar Nigeria; d Department of Chemical Sciences, Federal University of Wukari Wukari Taraba State Nigeria; e Department of Chemical Sciences, University of Johannesburg South Africa; f Centre for Herbal Pharmacology and Environmental Sustainability, Chettinad Hospital and Research Institute, Chettinad Academy of Research and Education Kelambakkam-603103 Tamil Nadu India

## Abstract

Retraction of ‘Yttrium- and zirconium-decorated Mg_12_O_12_–X (X = Y, Zr) nanoclusters as sensors for diazomethane (CH_2_N_2_) gas’ by Terkumbur E. Gber *et al.*, *RSC Adv.*, 2023, **13**, 25391–25407, https://doi.org/10.1039/D3RA02939E.

The Royal Society of Chemistry hereby wholly retracts this *RSC Advances* article due to concerns with the reliability of the data.

The noncovalent interactions (NCI) plots in Fig. 8 are identical to each other and to figures in another paper by the same author group but representing different systems.^1,2^ The authors have not been able to satisfactorily explain this.

In addition, a number of references were inappropriately replaced with self-citations by the authors during the revision process.

Given the significance of these concerns, the Editor has lost confidence that the findings presented in this paper are reliable.

The authors were informed but have not responded to any correspondence regarding the retraction.

Signed: Laura Fisher, Executive Editor, *RSC Advances*

Date: 28th March 2025

**References**

1. M. O. Odey, E. E. Antai, E. A. Adindu, O. C. Godfrey, I. U. Bassey, F. O. Nwaokolrie, A. Owolabi, A. Nkang, T. E. Gber, M. M. Edim and H. Louis, *Chem. Phys. Impact*, 2023, 100346.

2. T. E. Gber, H. Louis, O. C. Ngana, I. O. Amodu, E. E. Ekereke, I. Benjamin, S. A. Adalikwu and A. Adeyinka, *RSC Adv*., 2023, **13**, 25391–25407.

